# Caregiver experiences and observations of intrathecal idursulfase-IT treatment in a phase 2/3 trial in pediatric patients with neuronopathic mucopolysaccharidosis II

**DOI:** 10.1186/s13023-024-03034-y

**Published:** 2024-03-10

**Authors:** Karen S. Yee, Sandy Lewis, Emily Evans, Carla Romano, David Alexanderian

**Affiliations:** 1 Takeda Development Center Americas, Inc., Cambridge, MA USA; 2 RTI Health Solutions, Research Triangle Park, Raleigh, NC USA; 3 Takeda Development Center Americas, Inc., Lexington, MA USA

**Keywords:** Cognitive impairment, Mucopolysaccharidosis II, Hunter syndrome, Idursulfase, Intrathecal, Pediatric, Neuronopathic, Intellectual disability, Health-related quality of life, Patient-focused drug development

## Abstract

**Background:**

Approximately two-thirds of patients with mucopolysaccharidosis II (MPS II) have a severe, neuronopathic phenotype, characterized by somatic, cognitive, and behavioral issues. Current standard of care for the treatment of MPS II is enzyme replacement therapy with intravenous recombinant human iduronate-2-sulfatase (idursulfase). To target cognitive manifestations of MPS II, idursulfase has been formulated for intrathecal administration into the cerebrospinal fluid (idursulfase-IT). In accordance with recommendations for patient-focused drug development, semi-structured interviews were conducted to assess caregiver experiences and observations in a 52-week phase 2/3 trial of idursulfase-IT, in addition to intravenous idursulfase in pediatric patients with neuronopathic MPS II, or a substudy which enrolled patients younger than 3 years old, all of whom received idursulfase-IT.

**Results:**

Overall, 46 caregivers providing care for 50 children (mean [range] age 7.9 [3–17] years at interview) took part in a single 60-min exit interview; six of these children had participated in the substudy. Qualitative and quantitative data were obtained demonstrating the burden of MPS II experienced by caregivers and their families. Following participation in the trials, 39 (78%) of the children were reported by their caregivers to have experienced improvements in the symptoms and impact of disease. Of those with improvements, 37 (95%) experienced cognitive improvements and 26 (67%) experienced emotional/behavioral improvements. Overall, 43 children (86%) were rated by caregivers as having moderate or severe symptoms before the trials; after the trials, 28 children (56%) were considered to have mild or no symptoms. For the six children who participated in the substudy, these proportions were 83% and 100%, respectively. Caregivers’ qualitative descriptions of trial experiences suggested improvements in children’s verbal and non-verbal functioning and spatial and motor skills, as well as a positive impact on family life.

**Conclusions:**

This study revealed caregiver-reported improvements in children’s MPS II symptoms and the impact of the disease on patients and their families. There was a trend for cognitive improvement and a reduction in severity of MPS II symptoms. After many years of extensive review and regulatory discussions of idursulfase-IT, the clinical trial data were found to be insufficient to meet the evidentiary standard to support regulatory filings.

**Supplementary Information:**

The online version contains supplementary material available at 10.1186/s13023-024-03034-y.

## Background

The mucopolysaccharidoses are a group of 12 inherited metabolic diseases characterized by the absence of specific lysosomal enzymes needed to break down glycosaminoglycans (GAGs) [1, 2]. Mucopolysaccharidosis II (MPS II; Hunter syndrome; OMIM 309900) is the only X-linked MPS, mainly manifesting in male patients. It has an estimated birth prevalence of 0.1–2.16 per 100,000 live births [3, 4]. MPS II is characterized by a deficit in lysosomal iduronate-2-sulfatase, which leads to accumulation of two GAGs, heparan sulfate and dermatan sulfate, throughout the body. This results in a progressive, multisystemic, heterogeneous phenotype involving most organs and body systems. Signs and symptoms of MPS II typically appear at 2–4 years of age and include joint stiffness, skeletal deformities, coarsening of facial features, respiratory difficulties, cardiac abnormalities, hernias, and organomegaly [4, 5]. Approximately two-thirds of patients with MPS II have the severe, neuronopathic form of the disease [6], which is characterized by cognitive impairment and behavioral issues in addition to somatic manifestations [7, 8]. Respiratory obstruction and cardiac abnormalities are the primary causes of death in patients with MPS II [9], and patients with the neuronopathic form of the disease often die in the first two decades of life [4, 5, 10]. Neuronopathic MPS II is associated with a greater deficit in patient quality of life (QoL) than non-neuronopathic disease and also places substantial emotional, social, and financial burdens on caregivers and family members [7, 9, 11–14].

Disease-specific treatments for MPS II are in various stages of development. Intravenous (IV) enzyme replacement therapy (ERT) with recombinant human iduronate-2-sulfatase (idursulfase; Elaprase®, Takeda Pharmaceuticals USA, Inc., Lexington, MA, USA) is currently the only approved treatment by the US Food and Drug Administration (FDA) [15]; however, idursulfase beta and pabinafusp alfa are also available in some countries [16, 17]. Clinical studies and analyses of real-world data have shown that weekly IV idursulfase can stabilize or improve a range of somatic clinical parameters, including measures of pulmonary function, distance walked in the 6-min walk test, liver and spleen size, and urinary GAGs [18–20]. Analysis of real-world data has also demonstrated increased survival in patients receiving IV idursulfase treatment compared with untreated patients [21]. However, idursulfase is a large, glycosylated enzyme and, thus, is unable to cross the blood–brain barrier when administered intravenously [22]. Consequently, IV idursulfase does not have a direct impact on cognitive manifestations of MPS II [22, 23]. As such, several treatments have been investigated to attempt to treat neuronopathic MPS II, including ERTs designed to enter the central nervous system [24], gene therapy [25], and a formulation of idursulfase for direct administration into the cerebrospinal fluid (CSF) via an implanted intrathecal drug delivery device (IDDD) [26, 27].

The effects of monthly intrathecal (IT) idursulfase (idursulfase-IT; administered in addition to weekly IV idursulfase as the standard of care) in preventing cognitive decline and early cognitive impairment in children with MPS II have been evaluated in a phase 2/3 study (HGT-HIT-094; NCT02055118). Patients who completed this 52-week assessor-blinded study, or a separate open-label substudy of patients starting treatment before the age of 3 years, were subsequently enrolled in an open-label extension study (SHP609-302; NCT02412787; ongoing) in which all patients received monthly idursulfase-IT. The primary endpoint of the phase 2/3 study was cognitive function, assessed by change in Differential Ability Scales, Second Edition (DAS-II) [28] General Conceptual Ability (GCA) score from baseline to week 52. The key secondary endpoint was changes in the Vineland Adaptive Behavior Scales, Second Edition (VABS-II) Adaptive Behavior Composite (ABC) score [29]. The evidence after 3 years of follow-up supports a treatment effect for idursulfase-IT in patients who began idursulfase-IT treatment before 6 years of age, even though the phase 2/3 study did not meet its primary endpoint at 12 months [26, 27].

It is recognized that formal assessment of cognitive function in patients with diseases such as MPS II poses several major challenges owing to the physical and behavioral characteristics of this patient population [1]. In addition, there is no validated disease-specific tool for measuring cognitive and behavioral outcomes in patients with MPS II. There is growing recognition by all stakeholders, including the FDA, of the value of incorporating patient-relevant outcomes such as behavioral and social-emotional state, caregiver burden, and QoL into clinical trial design [1, 30–34]. A non-interventional study was therefore conducted following the phase 2/3 idursulfase-IT clinical trial to capture patient and family experiences and observations through structured caregiver exit interviews. The objective of this study was to gather additional insight via direct feedback from caregivers to facilitate interpretation of the trial data and to explore treatment benefits further.

## Methods

### Study overview

This was a prospective observational study consisting of a single in-depth, semi-structured telephone interview with caregivers of children who participated in the phase 2/3 idursulfase-IT clinical trial, including those in an associated substudy conducted in patients younger than 3 years of age.

### Phase 2/3 idursulfase-IT trial design

**Fig. 1:**
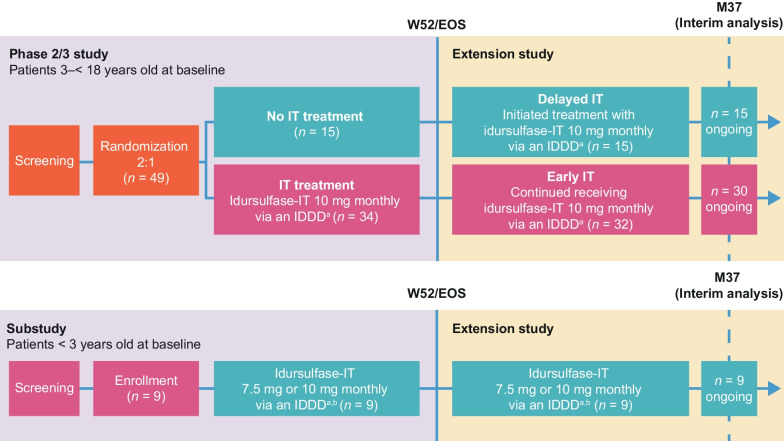
Overview of idursulfase-IT primary and extension study design [26, 27]. ^a^ Idursulfase-IT was administered via a surgically implanted IDDD (SOPH-A-PORT Mini S; Sophysa SA, Orsay, France). If the IT space was inaccessible, or in the event of device malfunction, idursulfase-IT could be administered by lumbar puncture. ^b^ Substudy patients aged > 8–30 months at dosing received an adjusted dose of idursulfase-IT 7.5 mg; patients aged > 30 months–3 years at dosing received idursulfase-IT 10 mg. *EOS* End of study, *IDDD* Intrathecal drug delivery device, *IT* Intrathecal, *M* Month, *W* Week

Study patients with MPS II and early cognitive impairment had previously received and tolerated a minimum of 4 months of therapy with IV idursulfase during the period immediately before screening. All participants continued to receive weekly IV idursulfase as standard of care. A substudy was conducted for children younger than 3 years of age in which the idursulfase-IT dose was adjusted (7.5 or 10 mg monthly) based on reference brain weight (Fig. 1).

Cognitive impairment was defined according to age, as follows: 3– < 13 years, DAS-II GCA score 55–85 or DAS-II GCA score > 85 plus ≥ 10-point decrease in GCA score over 12 months from a previously documented result in the observational study HGT-HIT-090 (NCT01822184) [35]. Key exclusion criteria were opening CSF pressure upon lumbar puncture > 30.0 cm H_2_O, and functioning CSF shunt device [26, 27].

Neurodevelopmental assessments were conducted at screening and at weeks 16, 28, 40, and 52 (end of trial). The primary efficacy endpoint was change in DAS-II GCA score from baseline to week 52. The key secondary endpoint was change in VABS-II ABC score from baseline to week 52.

Patients who completed the phase 2/3 study or substudy were enrolled in an extension study in which all patients received treatment with monthly idursulfase-IT in addition to weekly IV idursulfase [26, 27] (Fig. 1).

### Interview methodology

Clinical trial sites from Canada, France, Mexico, Spain, the UK, and the USA were invited to participate in this interview study; owing to implementation challenges, the clinical trial site in Australia (representing one patient with MPS II) was unable to participate. Participation was voluntary, and a monetary incentive (US$ 100) was given to the caregiver of the study patient regardless of the length of the interview.

Each caregiver provided informed consent to participate in this study. Within 2 days of obtaining consent, recruitment logs containing the caregiver’s unique study identification number, first name, phone number, sex, email address, and date of the child’s phase 2/3 idursulfase-IT trial end-of-treatment visit were completed. Interviews were conducted by phone by trained staff from RTI Health Solutions (Research Triangle Park, NC, USA) in the USA, UK, and Canada or approved RTI subcontractor, AplusA (Newark, NJ, USA) at all other sites, utilizing a semi-structured interview guide. Verbatim transcripts were prepared for analysis by trained RTI-Health Solutions staff. For caregivers who had multiple children in the clinical trial, the caregiver was asked to provide feedback on all children during a single interview.

The semi-structured interview lasted approximately 60 min and was designed to assess: (1) pre-trial signs and symptoms of MPS II observed by the caregiver in the idursulfase-IT trial participants; (2) the impact of MPS II on daily activities, social interactions, and behavior in both trial participants and caregivers before the trial; (3) caregivers’ pre-trial treatment expectations; and (4) the perceived changes in the symptoms and impact of disease in children during the trial.

### Data analysis

Using transcripts from audio recordings of the interviews, dominant trends were identified across interviews to generate themes or patterns in the way caregivers described their pre-treatment expectations, experiences, and observations before and during the clinical trial, and perceived treatment-related benefits observed over the trial period. All names mentioned during the interview were removed from the transcript. Descriptive summary statistics are provided for the studied outcomes as appropriate. Owing to the qualitative nature of the study, no formal statistical analyses were performed.

## Results

### Participants

Forty-nine patients aged 3– < 18 years with MPS II and early cognitive impairment were enrolled in the main phase 2/3 idursulfase-IT trial; 34 were randomized to receive monthly idursulfase-IT 10 mg and 15 to no IT treatment. Overall, 47 patients completed the study; two participants in the idursulfase-IT arm withdrew their consent. All patients in the substudy (*n* = 9) received idursulfase-IT, and all completed the substudy [26, 27].

For the interview study results presented herein, data were obtained from 46 caregivers who were providing care for a total of 50 children who participated in the phase 2/3 trial or substudy. Caregiver and trial participant demographics are presented in Table 1. Most caregivers (83%) were female, and the mean (range) age was 38.6 (27–50) years. Nearly two-thirds (63%) of participating caregivers were based in the USA. The mean (range) age of all children at diagnosis was 24.3 months (in utero–5 years), 4.0 (2–12) years at trial entry, and 7.9 (3–17) years at the time of interview. Six of the children had participated in the substudy; for this subset, the mean (range) age was 12.8 months (in utero–20 months) at diagnosis and 2 (2) years at trial entry.

**Table 1:** Caregiver and trial participant demographics

	Interviewed caregivers (*N* = 46)		Trial participants (*N* = 50)
**Sex, n (%)**			
Male	8 (17.4)		50 (100.0)
Female	38 (82.6)		0 (0.0)
**Age, mean (range)**			
Age at time of interview, years	38.6 (27–50)^a^		7.9 (3–17)
Age at diagnosis for all trial participants, months	–		24.3 (in utero–5 years)
Age at diagnosis for substudy participants, months	–		12.8 (in utero–20 months)
Age at trial entry for all trial participants,^b^ years	–		4.0 (2–12)
Age at trial entry for substudy participants, years	–		2 (2)
**Caregiver/participant country, n (%)**			
USA		29 (63.0)	
Spain		5 (10.9)	
Mexico		4 (8.7)	
Canada		3 (6.5)	
France		3 (6.5)	
UK		2 (4.3)	
**Race/ethnicity,**^**c**^** n (%)**			
White	32 (69.6)		29 (58.0)
Hispanic or Latino	10 (21.7)		10 (20.0)
Two or more races	4 (8.7)		7 (14.0)
Asian	3 (6.5)		3 (6.0)
Black	2 (4.3)		–
Other (not specified)	–		1 (2.4)
**Caregiver education,**^**d**^ **n (%)**			
High school or equivalent	6 (13.0)		–
Some college	8 (17.4)		–
Associate or technical degree	4 (8.7)		–
College degree	12 (26.1)		–
Postgraduate degree	9 (19.6)		–

^a^Not reported for 5 caregivers

^b^Data not available for participants outside the USA and Canada

^c^Not reported for patients in the UK and France (collecting these data in France is against regulations). Totals were greater than 50 because Hispanic or Latino was a separate question from race

^d^Not reported for 7 caregivers

### Diagnosis to pre-trial

**Fig. 2:**
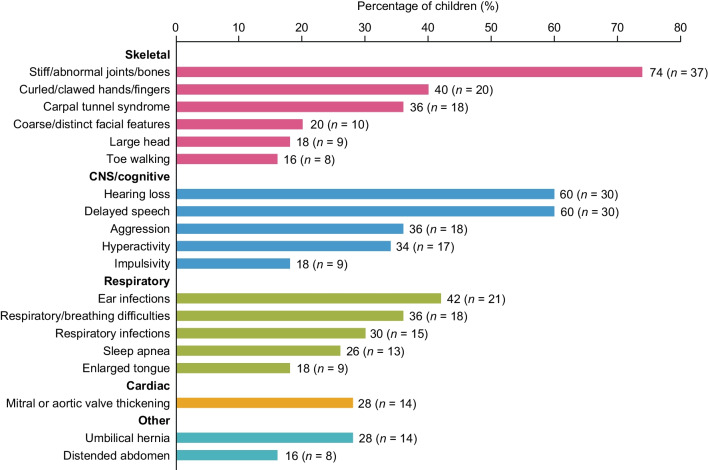
Caregiver-reported pre-trial MPS II signs and symptoms with a prevalence of ≥ 15%**.** *CNS* Central nervous system, *MPS* *II* Mucopolysaccharidosis II

Caregivers were asked to provide information on signs and symptoms before the diagnosis of MPS II in children participating in the idursulfase-IT trial. Frequent respiratory infections (36%), delayed speech (32%), hearing loss (28%), and ear infections (28%) were most frequently reported (Fig. S1 [Additional file 1]). Caregivers also reported signs and symptoms of MPS II before enrollment in the trial. The most frequently reported symptoms at this stage included stiff/abnormal joints/bones (74%), hearing loss (60%), delayed speech (60%), and ear infections (42%) (Fig. 2). Caregiver narratives relating to signs and symptoms of MPS II are presented in Table S1 [Additional file 2].

**Fig. 3:**
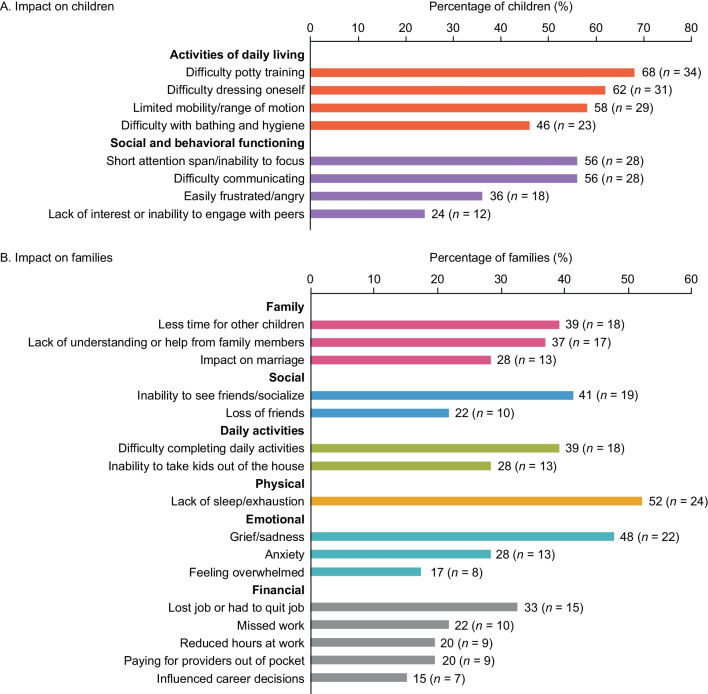
Caregiver-reported pre-trial impact of MPS II, with a prevalence of ≥ 15%. *MPS* *II,* Mucopolysaccharidosis II

Caregivers were asked to describe their observations of the detrimental impact of MPS II on the children before participating in the idursulfase-IT trial. Caregivers most frequently reported difficulty with potty training (68%), difficulty dressing oneself (62%), limited mobility/range of motion (58%), short attention span/inability to focus (56%), and difficulty communicating (56%) (Fig. 3A). In addition, caregivers described the impact of MPS II on themselves and their families. The most commonly reported effects were lack of sleep/exhaustion (52%), grief/sadness (48%), inability to see or socialize with friends (41%), having less time for other children (39%), difficulty completing daily activities (39%), lack of understanding or help from family members (37%), and employment changes (lost job/had to quit their job) (33%) (Fig. 3B). Caregiver narratives relating to the personal and family impact of MPS II are presented in Tables S2 [Additional file 3] and S3 [Additional file 4]. These demonstrate a devastating effect on caregivers’ personal and professional lives. For example: “*We have to be with him all the time, and everything that has to do with work, with our free time, we have left it aside, abandoned. But all the rest, our expectations, our hopes … everything is gone.” (child aged 8 years at time of interview)* and “*I’m basically unable to work. Because if I did work, then I’d just be paying a nurse to be here.” (aged 4 years at trial entry; aged 9 years at time of interview).*

### Caregivers’ expectations before the phase 2/3 idursulfase-IT trial

Caregivers were asked to recall their expectations before the initiation of their child’s treatment. Their responses suggested that they considered the idursulfase-IT trial the only option at the time to help to extend their children’s lives and to improve their QoL (Table S4 [Additional file 5]). These views were evident through narratives such as: “*We are trying everything and … anything and everything to save him. So, we’re taking any little glimpse of hope we can.*” *(aged 2 years at substudy entry; aged 3 years at time of interview)*; “*There is no trajectory for him except down, unless we try anything and everything.*” *(aged 3 years at trial entry; aged 7 years at time of interview)*; and “*It’s either do you want the possibility of extending your child’s life, or do you want the certainty of his death? That was it.*” *(aged 4 years at trial entry; aged 6 years at time of interview)*. Caregivers specifically desired to see improvements in their children’s cognitive functioning: “*I enrolled him because I could see my son somewhere buried into his body … like, his personality, I knew it was there and I knew he wanted to come out and show us who he really was, but the disease was sort of holding him back because it was affecting his brain.*” *(aged 6 years at time of interview)* and “*I want my child to be able to talk to me. I want him to live. It would allow him to be [himself]. It would allow him not to be shut behind those glass doors of his eyes. It would allow him just to have a voice longer … and that’s worth it*” *(aged 4 years at trial entry; aged 7 years at time of interview).*

### Experiences of the phase 2/3 idursulfase-IT trial: perceived changes in symptoms

Regarding perceived improvements during the phase 2/3 trial or substudy, caregivers’ feedback indicated that 39 children (78%) experienced an improvement in observable signs and symptoms (Table 2). Of these 39 children, 37 (95%) demonstrated caregiver-reported evidence of cognitive improvements, 26 (67%) showed emotional or behavioral improvements, 22 (56%) showed improvements in daily activities, 17 (44%) showed social improvements, 12 (31%) showed physical improvements, and 4 (10%) noted improvements in family dynamics. Specifically, the most reported improvements within the cognitive domain were in speech (21 children, 54%) and the ability to communicate needs or wants/engage in conversation (20 children, 51%).

**Table 2:** Caregiver-reported improvements in signs and symptoms and disease impact following participation in the idursulfase-IT trial^a^

Improvement	Children in the pivotal trial and substudy *N* = 39 *n* (%)	Children in the substudy *N* = 6 *n* (%)
**Cognitive improvements**	37 (94.9)	5 (83.3)
Improved speech	21 (53.8)	4 (66.7)
Improved ability to communicate needs or wants/engage in conversation	20 (51.3)	1 (16.7)
Continuing to gain skills	18 (46.2)	5 (83.3)
Improved recall and memory	9 (23.1)	–
Exhibiting higher-level thinking skills	9 (23.1)	–
**Emotional/behavioral improvements**	26 (66.7)	1 (16.7)
Improved ability to focus/longer attention span	13 (33.3)	1 (16.7)
Decreased aggression	12 (30.8)	1 (16.7)
Decreased hyperactivity	8 (20.5)	–
Improved mood stability	2 (5.1)	–
**Improvements in activities of daily living**	22 (56.4)	3 (50.0)
Improved potty training	13 (33.3)	2 (33.3)
Improved ability to dress oneself	8 (20.5)	3 (50.0)
Improved ability to bathe oneself or brush one’s teeth	5 (12.8)	1 (16.7)
Improved ability to feed oneself	2 (5.1)	–
**Social improvements**	17 (43.6)	–
**Physical improvements**	12 (30.8)	2 (33.3)
Improved balance	6 (15.4)	1 (16.7)
Improved mobility/decreased stiffness	5 (12.8)	1 (16.7)
Improved vision	2 (5.1)	–
**Improvements in family dynamics**	4 (10.3)	–

^a^Overall, improvements were observed in 39/50 children (78.0%). The percentage reported here was calculated based on the 39 children with reported improvements. For 11/50 children (22.0%), improvements were not observed; these children are not represented in this table

*IT* Intrathecal

In 11 children (22%; mean age [range]: 8.4 [7─10] years), caregivers reported no observable improvements during the idursulfase-IT trial. Of these, nine children were reported to have no or limited expressive language and continued to require assistance in most daily activities. Most of these children were also described as having behavioral symptoms, including hyperactivity, inattention, and/or aggression. Four caregivers reported worsening behavior such as increased displays of frustration or anger in their children after beginning the clinical trial. However, these behavioral changes were viewed as improvements by caregivers because they regarded them as evidence that their children were understanding more or deepening their engagement with the world.

Caregivers for the six children in the substudy reported improvements in disease signs and symptoms (Table 2). Cognitive improvements were reported in five of the children, including an improvement in the ability to gain new skills (*n* = 5) and in speech (*n* = 4). In terms of daily activities, three children improved their ability to dress themselves, while two children improved their potty training.

### Experiences of the phase 2/3 idursulfase-IT trial: perceived improvement in disease severity

**Fig. 4:**
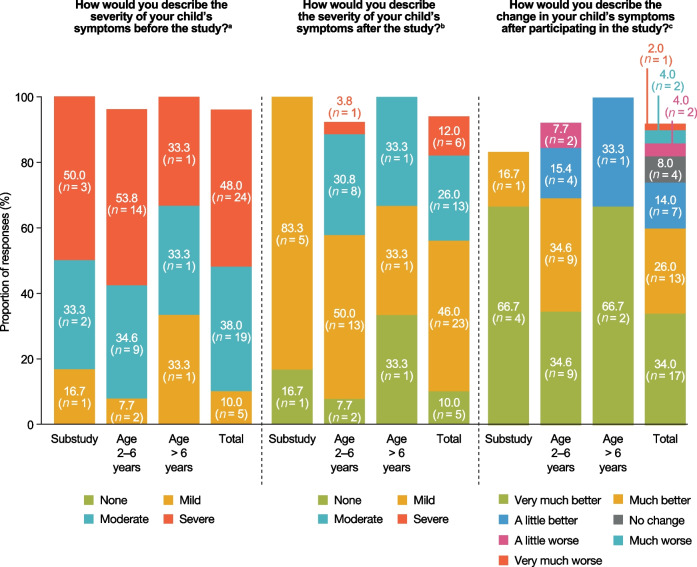
Perceived disease severity before and after the idursulfase-IT trial based on caregivers’ feedback. Only the total column reflects all interviews; age at study entry was not available for children outside the USA and Canada. ^a^ One participant whose child was in the age 2–6 years category was unable to provide a response to this question; one participant whose child’s age at study entry was unavailable was unable to provide a response to this question. ^b^ Two participants whose children were in the age 2–6 years category were unable to provide responses to this question; one participant whose child’s age at study entry was unavailable was unable to provide a response to this question. ^c^ Three participants, two of whom had children in the age 2–6 years category and one of whom had a child in the substudy, were unable to provide a response to this question; one participant whose child’s age at study entry was unavailable was unable to provide a response to this question. *IT* Intrathecal

Before the idursulfase-IT trial, 43 children (86%) in the phase 2/3 trial or substudy presented moderate or severe disease symptoms based on caregivers’ perceptions (Fig. 4). Fewer children were categorized with moderate or severe disease symptoms (19; 38%) following the trial, when 28 children (56%) were instead rated by caregivers as having mild or no symptoms. Six children (12%) were rated as having ongoing severe symptoms after the trial. In the substudy group, five of the six children presented moderate or severe symptoms before the trial based on caregivers’ perceptions. After the trial, all six children were rated as having mild or no symptoms. After the idursulfase-IT trial, 30 children (60%) overall (including the six children in the substudy) were rated as having ‘very much better’ or ‘much better’ disease symptoms compared with symptoms before the study (Fig. 4).

### Experiences of the phase 2/3 idursulfase-IT trial: qualitative narratives

Caregiver narratives describing observed improvements following the children’s participation in the idursulfase-IT trial are shown in Table 3. Observations have been categorized under themes relevant to the outcome scales used in the trial. For example, improvements were described in verbal skills: “*He just started exploding, like 6 months into the study, he just started exploding into words. And he started picking up words, and then it just progressed from there. Then he got two-word sentences. Then he got three-word sentences. Then he got four-word sentences.” (aged 4 years at trial entry; aged 7 years at time of interview)*. Improvements were also reported in non-verbal skills: “*He understands more of, you know, cause and effect.” (aged 4 years at trial entry; aged 9 years at time of interview).* Caregivers described development in spatial skills (“*And he knows when he went in the hotel, he knows what room. He don’t have to read, but he remembers which direction, go left or right.” [aged 4 years at trial entry; aged 6 years at time of interview]*) and motor skills (“*I mean the two big things that we noticed after only a couple of doses was that he was a lot more able to do things. Climbing, like he never climbed up on anything to the extent that he does now anyways. Like, he can get up on a chair maybe, but he’s able to climb up on things. He’s a lot faster when he [runs].” [aged 2 years at trial (substudy) entry; aged 5 years at time of interview]*). There was also evidence of improvements in skills associated with daily living (“*He can help put his shirts on more. He can pull off his pants. He can pull up his pants if I put his feet in his pants. He can take his own socks off … Certain shoes he can put on. Same with when he’s bathing. He will put soap on and he tries … He knows to use a toothbrush to run the bristles along his teeth … So, there’s lots of skills that he’s gained due to being on the trial.” [aged 3 years at trial entry; aged 7 years at time of interview]*) and socialization (“*The aggressiveness has decreased a lot, and about hyperactiveness, well, I will take him to [the] park nearby, and he will play there as a normal kid.” [aged 9 years at time of interview]*).

**Table 3:** Caregiver narratives of observations following participation in the idursulfase-IT trial

Narrative	Patient age at trial entry/time of interview, years
**Verbal**	
*He would say ‘vizza,’ and all of a sudden it became ‘pizza.’ And then ‘nanna’ became ‘Hannah’ just after that first dose. It was amazing. I was like ‘Seriously?’ [laughter] I haven’t seen drastic changes on a month-to-month, but I do see that he’s still learning. But after just that first dose, I was convinced*	NA/8
*His speech, 2 weeks after his first dose, he said the word—and I have the video—'dinosaur.’ Clear as day. Clear as day as he was playing with his dinosaurs on the couch. Clear as day. I still, it brings me to tears. [crying] [My son] can speak words. He can say the full word*	3/7
*He just started exploding, like 6 months into the study, he just started exploding into words. And he started picking up words, and then it just progressed from there. Then he got two-word sentences. Then he got three-word sentences. Then he got four-word sentences*	4/7
*Improvements in [my child] … since starting the study, he comprehends everything*	7/12
**Non-verbal**	
*The first thing is that he is doing very well in school. He is more interested in mathematics than in writing or reading*	NA/9
*He understands more of, you know, cause and effect*	4/9
*The other thing we noticed is that in terms of connection, there was something where we were in the car and he could say like ‘green light.’ He would always say when the light turned green, ‘green light, green light.’ This was before IT. After IT, just spontaneously, you know, and this was only a couple of treatments in, maybe 10 treatments, in he goes, ‘Mommy, that’s a green arrow. We go left.’ He was making brain connections. He was putting two things together that I had never seen him put together*	4/6
*He knows boundaries, so we’ll tell him, ‘Okay you got 5 min, and then, you got to go to bed’ or ‘You got 5 min and then, you got to turn the TV off.’ And he actually understands that. He understands the concept of time*	NA/4
**Spatial**	
*He’s counting by 10*	2/5^a^
*But now, like I can sit him down. I’ll explain and ‘if/then,’ and he might argue with me or it might take a while, but like he gets around to it, and he understands the ‘if/then’ scenario, and he can make a choice based on that. So, there’s a lot of reasoning and a lot of just, I don’t know, like … the fog being cleared, if that makes any sense*	3/7
*When the doctor says, ‘Open up,’ he’ll say ‘sayahh.’*	3/7
*And he knows when he went in the hotel, he knows what room. He don’t have to read, but he remembers which direction, go left or right*	4/6
*His ability to [connect] concepts like ‘That shirt goes on my head. Put the shirt on my head.’ [Or] ‘People are married. They get married and [have] kids.’*	4/7
*If you took apart a motor, he could probably help you put it back together … he’s got that, almost like a photographic memory when it comes to those things*	4/8
**Daily living skills**	
*The first thing that happened that we were shocked by was that he potty trained. He wasn’t potty trained before or, you know, like he just didn’t pay attention, wasn’t, you know, could not put two and two together. And it was a few months after a few doses, it’s like something just clicked*	3/7
*He can help put his shirts on more. He can pull off his pants. He can pull up his pants if I put his feet in his pants. He can take his own socks off … Certain shoes he can put on. Same with when he’s bathing. He will put soap on and he tries … He knows to use a toothbrush to run the bristles along his teeth … So, there’s lots of skills that he’s gained due to being on the trial*	3/7
*He, I mean, he’ll completely dress himself. Which, I mean, I knew he’d be [inaudible]. He did before. But now it’s shoes and socks and I can just, basically I can hand him the clothes and say, ‘Okay, it’s time to get dressed for school,’ and he’ll put them on without me having to go in there and like prompt him to do things*	4/9
**Socialization**	
*The aggressiveness has decreased a lot, and about hyperactiveness, well, I will take him to [the] park nearby, and he will play there as a normal kid*	NA/9
*Social. I’d say very caring of his other classmates. He’s very cognizant of other people’s situation[s]. Yeah. I mean, he’s, yeah, he’s lovely, and everyone is in love with him*	3/7
*He’s a very social little boy. He likes to take people’s hands and show them things and tries his best to communicate as best as he can. Completely engages. And appropriately too*	3/7
*Now, he taps friends on the shoulder, and he says, ‘Can I play with you?’ Now, he might still be or he might come and tap them and say, ‘Let’s play duck-duck-goose.’ Now he still has a problem if the person says no. He might repeat, ‘Let’s play duck-duck-goose.’ But he’s socially much more appropriate. He can engage in a board game like Candy Land*	4/6
**Motor skills**	
*I mean the two big things that we noticed after only a couple of doses was that he was a lot more able to do things. Climbing, like he never climbed up on anything to the extent that he does now anyways. Like, he can get up on a chair maybe, but he’s able to climb up on things. He’s a lot faster when he [runs]*	2/5^a^
*We turned around and in terms of eye-hand coordination; yeah, he was off the chart on the scoreboard*	3/7
**Family life**	
*We were taking vacations solo. [My husband] would go, and then I would go or whatnot, and uh … last year, we actually went as a family and it was an amazing vacation. Like I was surprised. I was actually dreading going on this vacation because I just wasn’t sure how it would go because [my husband] and I had avoided it for so many years*	4/10
*I think the biggest thing for us was going from where we were functioning, you know, we were just coping … Our family as a whole, we’re able to function much, much better. Much more peace, much more happy, the quality of our family’s life and our ability to care for not just [our child with Hunter syndrome], but all of our children was so much better*	5/9
*Taking him to the grocery store and actually going shopping with him. If we have a list, then we go over, then we get things on the list. You’re going to walk by my side. And so just that alone is huge. So, bringing him more into the community where he can act appropriately in some other social settings*	NA/8
*You can plan activities. You can, like you can socialize more with other families. Everything’s just a little bit brighter. His behavior has improved to the point that it is more socially acceptable than it was. And so people are more tolerant of him. And so it’s not nearly as isolating as it was*	7/11

^a^Patient enrolled in substudy

*IT* Intrathecal, *NA* Not available

Caregivers also described the impact of their child’s participation in the trial on themselves and their families (Table 3). For example, caregivers reported: “*Our family as a whole, we’re able to function much, much better. Much more peace, much more happy, the quality of our family’s life and our ability to care for not just [our child with Hunter syndrome], but all of our children was so much better.” (aged 5 years at trial entry; aged 9 years at time of interview)* and “*You can, like you can plan activities. You can socialize more with other families. Everything’s just a little bit brighter. His behavior has improved to the point that it is more socially acceptable than it was. And so people are more tolerant of him. And so it’s not nearly as isolating as it was.” (child aged 7 years at trial entry; aged 11 years at time of interview)*.

## Discussion

The feedback from caregivers of participants in the phase 2/3 trial of idursulfase-IT and the associated substudy demonstrated the substantial pre-trial burden of MPS II on the daily lives of children, caregivers, and families, and provided insight into caregivers’ perceptions of treatment effect on their children. After the trial, the number of children reported by caregivers to have moderate or severe symptoms decreased and the number of children with mild or no symptoms increased. Improvements in verbal and non-verbal functioning and in personal and social functioning were frequently described by the caregivers, together with an associated positive impact on emotional and behavioral functioning, and aspects of daily living for the children, caregivers, and family.

Caregiver descriptions of pre-trial disease burden are consistent with previously published reports of the somatic and neurological signs and symptoms of MPS II, and the impact of these on patients and families in terms of limitations and symptoms experienced by the patient, healthcare resource utilization, and effect on their daily lives [5, 14, 36]. In particular, caregivers reported in this study that children with MPS II experienced difficulty with potty training, dressing, limited mobility, short attention span, and difficulty communicating. The challenging behaviors associated with neuronopathic MPS II, which also include sleep disturbance, hyperactivity, agitation, aggression, and repeated behaviors, have a particularly strong impact on the lives of caregivers and families of patients with MPS II [8, 14, 37].

Based on caregiver reports, more than three-quarters of the children experienced some degree of improvement in disease symptoms following the idursulfase-IT phase 2/3 trial. Furthermore, the proportion of children experiencing moderate or severe disease symptoms during the trial more than halved (from 86 to 38%) according to their caregivers. Five of the six children in the substudy presented with moderate or severe symptoms before the trial, and yet were reported to have mild or no symptoms at all by the end of the trial; all six of these children received idursulfase-IT. Findings from the caregiver interviews indicated cognitive improvements in 95% of children who reported an improvement in symptoms, including improvements in speech and communication in around half of the children. The emotional impact of the improvements on caregivers was evident through interview responses such as: “*His speech, 2 weeks after his first dose, he said the word—and I have the video—'dinosaur.’ Clear as day. Clear as day as he was playing with his dinosaurs on the couch. Clear as day. I still, it brings me to tears. [crying] [My son] can speak words.” (child aged 3 years at trial entry, aged 7 years at interview).*

Behavioral improvements were also reported in almost two-thirds of the children. Although some caregivers reported a worsening in behavior after starting the trial, they interpreted the increased frustration as an improvement, because it showed that the children may in fact have been engaging better with their surroundings than before the trial. A study aiming to characterize neurobehavioral symptoms in neuronopathic MPS II has highlighted the propensity for observers to misinterpret an affected child’s attempts at social interaction or sensory-seeking activities, for example, as aggressive or inconsiderate behavior [8]. While caregivers will likely be more sensitive than others to the true nature of their child’s behavior, this nonetheless demonstrates the ease with which changes may be misconstrued. Further, this work highlights the importance of incorporating patient experience data into drug development. The nuanced perspective on the apparent worsening of behavior could be overlooked in standard clinical trial designs. This aligns with the latest FDA guidance that encourages stakeholders to collect such data to support product development and regulatory decision-making [31–34].

The impact of progressive somatic and neurological disease on QoL is substantial not just for children with MPS II but, as shown here and by other studies, also for those providing care [14, 38]. Notwithstanding the rewards associated with caregiving, it is common for caregivers to experience significant strain linked to finances, family conflict, loss of freedom, limited time for self-care, and loss of general health, and they may have to reduce work hours or stop working altogether [14, 39, 40]. Our findings highlight that the factors intrinsically linked to providing the care needed for children with MPS II, such as lack of sleep, sadness, and the inability to socialize and maintain a job, had a substantial effect on the lives of caregivers and their families. Based on the feedback from their interviews, caregivers clearly expressed an improvement in their QoL following the idursulfase-IT trial. Caregivers reported that home life was often more peaceful and that they could take part in typical activities again, as well as the reported improvements in control over disease symptoms in their children.

There is a growing emphasis on patient-focused outcomes in drug development and studies of rare diseases. Coupled with recognition of the challenges of formal cognitive testing in patients with diseases such as MPS II, this provided a strong rationale for evaluating caregiver feedback following the phase 2/3 idursulfase-IT trial and the associated substudy, in which the primary endpoint was formal assessment of change in cognitive function [1, 26, 27, 31–34, 41]. The semi-structured exit interview approach used in this study is in line with methodological patient-focused drug development guidance documents developed by the FDA [32]. The majority of caregivers were found to be willing to participate and demonstrated high levels of engagement with the study. Nonetheless, it is important to acknowledge the limitations of this study. It was not possible to link individual caregiver feedback to the trial, treatment received (except in the substudy, in which all patients received treatment), or trial withdrawals, limiting the conclusions that can be drawn. Of note, the idursulfase-IT trial was assessor-blinded owing to the nature of the intervention (IDDD implantation), and so there is a possibility that caregiver knowledge of treatment allocation may have influenced the responses provided.

General limitations inherent to a qualitative analysis are also applicable to this study; interview reporting is subjective and prone to recollection bias, and qualitative work lacks formal statistical analysis. In particular, at the time of interview, children were participating in the extension study, and it is possible that experience during that study may have influenced the recall of caregivers for their thoughts immediately following the main phase 2/3 trial or substudy. Related to this, there was also variation in the timing of the interview and therefore in the duration of time left in the extension study at this point. It is also worth noting the high level of anticipation demonstrated by the caregivers in terms of their recalled expectations of trial treatment. Caregivers reported entering their children into the clinical trial both because they wanted to extend their children’s lives and improve their QoL, and because they saw the idursulfase-IT trial as their only option. As the focus of the interviews was on improvements rather than changes, there may be an element of bias in reporting; however, the quotes presented were representative of the caregivers’ responses. In addition, approximately 70% of caregivers were white and 46% were educated to college degree level or higher, indicating that these results may not be generalizable to a wider patient population.

Despite these limitations, the caregiver narratives describing important and meaningful improvements observed following participation in the idursulfase-IT phase 2/3 trial and substudy provide valuable, additional qualitative information on the study outcomes and the impact of idursulfase-IT on cognitive impairment in MPS II, particularly in this population of patients with behavioral and physical limitations and for whom there is no validated, disease-specific tool [1]. Further, input from caregivers is a valuable asset that can help guide the selection of measurement tools in clinical trials for patients with neuronopathic MPS II, or help with the development of such tools that better align with caregiver perspective on treatment benefit.

## Conclusions

This study revealed caregivers’ experiences of the symptoms and burden of MPS II before diagnosis and before and after the idursulfase-IT phase 2/3 trial and associated substudy. The use of patient-focused outcomes assessed via interview is concordant with recommendations for rare disease clinical trials. Caregiver reports clearly documented improvements in symptoms after enrollment in the trial and provided evidence of a tendency for the severity of symptoms to decrease, particularly in the substudy patients, all of whom had received idursulfase-IT. Caregivers were able to recognize and to describe cognitive improvements in verbal and non-verbal functioning, and improvements in personal and social functioning after starting the trial. The caregivers also detailed how these changes had contributed to improved emotional and behavioral functioning in the children and improved daily life for the children, the caregivers, and the family unit.

However, after many years of extensive review and regulatory discussions, the data were found to be insufficient to meet the evidentiary standard to support regulatory filings. Idursulfase-IT will continue to be made available to patients who are currently enrolled in the ongoing open-label extension studies until an alternative approved treatment option is available to address cognitive symptoms. Nevertheless, this analysis demonstrates the need for the appropriate treatment of children with MPS II and cognitive impairment and the potential benefits such treatment can bring to the lives of patients and their families.

### Supplementary Information

**Additional file 1. Fig. S1. **Caregiver-reported signs and symptoms of MPS II before diagnosis

**Additional file 2. Table S1.** Caregiver descriptions of MPS II symptoms

**Additional file 3. Table S2.** Caregiver descriptions of MPS II impacts: personal

**Additional file 4. Table S3.** Caregiver descriptions of MPS II impacts: family

**Additional file 5. Table S4.** Caregiver descriptions of clinical trial expectations

## Data Availability

De-identified individual participant data from this particular report will not be shared as there is a reasonable likelihood that individual patients could be re-identified (owing to the limited number of study participants and the narratives provided).

## Abbreviations

ABC: Adaptive Behavior Composite
CSF: Cerebrospinal fluid
DAS-II: Differential Ability Scales, Second Edition
ERT: Enzyme replacement therapy
EOS: End of study
FDA: US Food and Drug Administration
GAG: Glycosaminoglycan
GCA: General Conceptual Ability
IDDD: Intrathecal drug delivery device
IT: Intrathecal
IV: Intravenous
M: Month
MPS II: Mucopolysaccharidosis II
NA: Not available
QoL: Quality of life
VABS-II: Vineland Adaptive Behavior Scales, Second Edition
W: Week
