# Supplementary material for: Caregiver experiences and observations of intrathecal idursulfase-IT treatment in a phase 2/3 trial in pediatric patients with neuronopathic mucopolysaccharidosis II
Source: Orphanet J Rare Dis. 2024 Mar 10;19:110. doi: 10.1186/s13023-024-03034-y (PMC10926613; doi:10.1186/s13023-024-03034-y)
Supplement: Supplementary file 2 — Additional file 2. Table S1. Caregiver descriptions of MPS II symptoms [file 13023_2024_3034_MOESM2_ESM.docx]

**Caregiver experiences and observations of intrathecal idursulfase-IT treatment in a phase 2/3 trial in pediatric patients with neuronopathic mucopolysaccharidosis II**

**Karen S. Yee, Sandy Lewis, Emily Evans, Carla Romano, David Alexanderian**

**Table S1.** Caregiver descriptions of MPS II symptoms

| **Narrative** | **Patient age at trial entry/time of interview, years** |
| --- | --- |
| Respiratory symptoms | |
| *He does not breath[e] like a normal child, of course; that is, he gets tired very easily, as soon as he runs a bit. And [he] feels tired and he frequently snores.* | NA/8 |
| *He’s had two [pressure equalization] tube surgeries, a tonsillectomy, and an adenoidectomy. His tonsils were huge, his adenoids as well.* | 4/6 |
| *He was more asthmatic. Fluid in his ears. It was in his airway a lot … It produced a lot of mucus for him.* | 4/7 |
| *His work of breathing and the noisy breathing … At around age 3, I think it was, we went through three bouts of pneumonia in one winter. It must have been two the one winter, one the following winter.* | 7/11 |
| Cardiac symptoms | |
| *He had what they call a mild aortic stenosis. It’s something that the doctor said that if we hadn’t been poking around in there, that we may have never known that it was there. And it’s been remained unchanged since diagnosis.* | 2/5^a^ |
| *He has a slight valve leak like regurgitation. But there are no limitations or anything.* | 4/7 |
| Skeletal symptoms | |
| *His arms had the typical MPS and they still do. Deformities in his ribs where he doesn’t have that flexion of it. His legs, so it’s very difficult for him to … he still runs awkwardly but he can run now.* | NA/7 |
| *He’s got like the thick eyebrows and the low nasal bridge and the prominent forehead and the large head. And his fingers are really curved.* | 3/7 |
| *He can’t bend his arms completely straight. Um, he has mild scoliosis. He had a tibial bone that was significantly turned out.* | 12/17 |
| Central nervous system/cognitive symptoms | |
| *He’s not expressive. He doesn’t know how to communicate back to you. But he understands everything you say.* | 2/3^a^ |
| *He didn’t fall over but he was like … he didn’t feel the balance, like he did not like steps. Um, he was very cautious.* | 2/3^a^ |
| *He did complain of headaches. He would just hold his head and say that it hurt … probably once or twice a month.* | 4/8 |
| *His mom at the time basically noticed that his [inaudible] word and sound development wasn’t progressing kind of where she was expecting it to be.* | NA/4 |
| *He’s very active and cannot sit still, [he] just take off and he [gets] angry [easily]. He still sometimes screams and yell[s] for no reason.* | NA/4 |

^a^ Patient enrolled in substudy

*MPS II* mucopolysaccharidosis II; *NA* not available
