# Supplementary material for: Caregiver experiences and observations of intrathecal idursulfase-IT treatment in a phase 2/3 trial in pediatric patients with neuronopathic mucopolysaccharidosis II
Source: Orphanet J Rare Dis. 2024 Mar 10;19:110. doi: 10.1186/s13023-024-03034-y (PMC10926613; doi:10.1186/s13023-024-03034-y)
Supplement: Supplementary file 3 — Additional file 3. Table S2. Caregiver descriptions of MPS II impacts: personal [file 13023_2024_3034_MOESM3_ESM.docx]

**Caregiver experiences and observations of intrathecal idursulfase-IT treatment in a phase 2/3 trial in pediatric patients with neuronopathic mucopolysaccharidosis II**

**Karen S. Yee, Sandy Lewis, Emily Evans, Carla Romano, David Alexanderian**

**Table S2.** Caregiver descriptions of MPS II impacts: personal

| **Narrative** | **Patient age at trial entry/time of interview, years** |
| --- | --- |
| Activities of daily living | |
| *He couldn’t put on his clothes, he couldn’t button his shirt or his pants because of his hands. His motor skills were diminished, and his swollen stomach made it hard for him to bend, and, tying his shoes, for example, was very hard … Maybe going to the toilet. He learned it easily at first, but then was too complicated, like he had never learned how. He was always peeing himself. That one is the most notorious, he was always peeing himself, and the fact that it was difficult for him to change his clothes.* | NA/7 |
| *So, before IT, he was in pull-ups still. Didn’t understand using the bathroom. He completely undresses himself. He actually prefers to not wear any clothes.* | 4/9 |
| *There were also things that seemed very difficult or close to impossible to do, like potty training. It just was never, before IT, it was never truly consistent. I mean there would be, he was getting there, but there would be several accidents a week, and it just didn't quite click or connect.* | 4/6 |
| *He couldn’t put on his clothes, he couldn’t button his shirt or his pants because of his hands.* | NA/7 |
| Emotional/behavioral functioning | |
| *He does have a short attention span. Sometimes I’m not quite sure if it’s just because he is not interested, like school stuff. I think that sometimes during the day, that is more of a factor for him, reasoning-wise, why he’s not going to concentrate. But he definitely has got less of an attention span than an average child.* | 2/3^a^ |
| *He’s very hyperactive. Like, for example a little bit aggressive. He shouts a lot, he wasn’t calm at any time, he was always running, screaming, and shouting all the time.* | NA/7 |
| *Constantly hitting, constantly biting. Just complete frustration that he would just start screaming. And then I go back to developmental age. Again, I truly think that he’s around a 3-year-old mentally because I’ve noticed that some of that has kind of gone away or at least calmed down a lot. He does have a lot of sensory issues and he’s in therapy for those. He loves to lick, like, everything and it’s weird.* | 3/5 |
| *He would run out in the road. He almost got hit by cars because he’d just run. There was no concept of danger.* | 4/9 |
| Social functioning | |
| *He wants to play with his peers. He loves being around other kids. The limitation that we have with the social is he’s still in that 3 to 5 age range where he parallel plays. He’s not interacting, so one of like, his goal right now, in school and helping everybody, is to get him to interact with his peers at a more appropriate level.* | NA/8 |
| *When he was very, very young, I took him to a play group, and the people that ran the group tried to engage with him regarding playing with toys, and he had no interest. And they told me that was not normal.* | 2/5^a^ |
| Cognitive functioning | |
| *And then before actually starting the medication, he had lost the words that he had, verbal words.* | NA/7 |
| *He’s 8 and he’s probably somewhere between a 3- and 5-year-old cognition-wise. So, he developed pretty typically, except for some of the speech, until he was about 3, in terms of cognitive speech. He kind of had been maintaining, maintaining, and then I think, once we got to about 6, I definitely noticed he started kind of learning at a slower pace than other kids.* | NA/8 |
| *He cannot understand what he is being told, he doesn’t speak, he doesn’t … doesn’t express himself.* | NA/8 |
| *He had probably about 50 words by the time he was about the age of 4 and most of those were words that, you know, he would be asking for specific food or asking for specific things and usually were just one word.* | 4/9 |

^a^ Patient enrolled in substudy

*IT* intrathecal; *MPS II* mucopolysaccharidosis II; *NA* not available
