# Supplementary material for: Caregiver experiences and observations of intrathecal idursulfase-IT treatment in a phase 2/3 trial in pediatric patients with neuronopathic mucopolysaccharidosis II
Source: Orphanet J Rare Dis. 2024 Mar 10;19:110. doi: 10.1186/s13023-024-03034-y (PMC10926613; doi:10.1186/s13023-024-03034-y)
Supplement: Supplementary file 4 — Additional file 4. Table S3. Caregiver descriptions of MPS II impacts: family [file 13023_2024_3034_MOESM4_ESM.docx]

**Caregiver experiences and observations of intrathecal idursulfase-IT treatment in a phase 2/3 trial in pediatric patients with neuronopathic mucopolysaccharidosis II**

**Karen S. Yee, Sandy Lewis, Emily Evans, Carla Romano, David Alexanderian**

**Table S3.** Caregiver descriptions of MPS II impacts: family

| **Narrative** | **Patient age at trial entry/time of interview, years** |
| --- | --- |
| Family – overall | |
| *Well, the impact has been enormous, huge; it changes your life completely. Everything that you had thought, all your plans, go to waste and now we only live focused on the child and for the child. We have to be with him all the time, and everything that has to do with work, with our free time, we have left it aside, abandoned. But all the rest, our expectations, our hopes … everything is gone.* | NA/8 |
| Family dynamics | |
| *Nobody in my family has ever been special needs, like, any of our generations. So I just didn't feel like they understood it. But it's easier for me to cope with it just not even seeing them. I don't like the feeling of being judged.* | NA/6 |
| *There was a lot of rejection from our close family. A lot of … we actually got more support from our friends than our family. Our family just didn’t understand, or didn’t want to understand. Firstly, anyone around us with children refused to come and see us anymore because they were afraid their children would catch the disease.* | NA/11 |
| *Well, his father and I split up, so yes, there was a clear impact. We split up.* | NA/12 |
| *A lot of times, I’m kind of ignoring behavior with other kids or ignoring some other things because I need to address [my child with MPS II]. We don’t go to a lot of things outside the home, mainly because of how [my child] will react or something like that, which is not such a big deal for the younger kids, but it’s becoming a bigger deal for the older kids.* | 3/5 |
| *I’m not saying it takes away all the attention from that kid [sibling without MPS II], but things are different. The dynamics are definitely different. The kid could feel a certain way, left out or whatnot.* | 2/3^a^ |
| *To this day, my husband’s family, his mom and dad still don’t really understand what’s going on [laughter] despite many tries of explaining it to them.* | 2/5^a^ |
| *They don’t understand him. I don’t like the fact that they don’t understand him. It’s just hard to even be around them because I know what they’re thinking.* | 3/5 |
| *It’s affected, I mean, personal relationships, like me and his mother. We have to work. We work nightshifts, so we have to work opposite. So, we never see each other, period. Because if one of us aren’t working, we’re in appointments.* | 2/3^a^ |
| *I don’t think you can be told your son has an incurable, life-limiting disease without that having an impact on the relationship. I mean it’s the most brutal thing that’s ever happened to us as a couple.* | 4/6 |
| Social functioning | |
| *We’ve lost all of our friends. People just don’t understand.* | 2/3^a^ |
| *So socially, yes, no more work so no relationships outside the home to breathe a bit. We don’t have that anymore, we’re together 24/7, so it’s a bit tiring. Day and night, it’s always mummy.* | NA/11 |
| *Parents, at some point, although it’s probably unsaid, really don’t want their children around another child who, you know, might be impulsive. So, the invitations for social functions and things like that lessen. And so there are only a few key people that really will make an effort to still socialize.* | 4/6 |
| Daily activities | |
| *It changes your life completely in all senses. All or almost all our activities turn around him, and it conditions everything you do—I don’t know—from your holidays to everything!* | NA/8 |
| *You basically have to work from the moment that you wake up [laughter] until the last moment that you go to bed.* | 6/10 |
| Emotional functioning | |
| *At the beginning, it was very hard, because we learned from the Internet about everything that could happen, all the problems, the life expectancy rate supposedly, and it was very difficult for us. Being alone is even more hard, because you have no one to talk about it [with]. Watching them and not knowing what to do, it's hard.* | NA/9 |
| *… looking back, it was absolutely horrendous, I’ve never … you know, we went almost 2 years of being parents thinking that our child was going to grow up and … you know, go to college, get a car, a wife and children and all those things and for it to literally be took away from you it was extremely traumatic and … I never thought we could get over it, I didn’t think we’d fully ever get over it* | NA/7 |
| *Overwhelmed in the sense that … Let’s see … I guess feeling helpless, like you know, what else can I do besides getting a degree in genetics.* | 3/11 |
| *I think there was a lot of anxiety in the beginning for me. For my wife, there was a lot of denial.* | 4/7 |
| *Every day was a battle to get through and to just manage through the day without anyone getting hurt or anyone getting badly hurt. I was angry. So angry and frustrated all the time … felt like a complete failure. It was incredibly isolating and frustrating and … at times, the anger was just overwhelming. And at times I just wanted to get in my car and drive away.* | 7/11 |
| Physical functioning | |
| *In the beginning, I went through a period of insomnia when he was first diagnosed. I didn't sleep properly for a very long time. I developed fibromyalgia.* | NA/6 |
| *Energy, what’s that? [Laughter] I am always exhausted.* | 3/7 |
| *I definitely don’t go to the doctor as much as I should [laughter] because I don’t have the time.* | 4/8 |
| *There was a point where I stopped working and stopped driving completely because I didn’t feel that I was safe on the road from the lack of sleep.* | 7/11 |
| Finances | |
| *It has been critical, because before, I had a good income, but I worked too many hours. It was like slavery. And now we only make enough to eat. Sometimes for the electricity bills or water bills, I have to save money. But the situation is critical, because it's not enough.* | NA/9 |
| *I don’t want everything to revolve around my work, but it’s a real problem anyways: I can feel my ability … not intellectually, but in terms of the attention I can give at work … it’s reducing. And that’s definitely linked to tiredness and possibly even to depression.* | NA/12 |
| *My husband works out of town, so he is not home very often, because I had to stop working because [my child] was not able to do full days at school … I had to quit my job and my husband had to go find a better paying job, which means working out of town 20 days of the month.* | 3/7 |
| *I’m basically unable to work. Because if I did work, then I’d just be paying a nurse to be here. Neither of my boys go to school full-time … But at times, my husband is working three jobs to help offset costs of things that we’ve had to do in order to provide for our kids and make sure that they have what they need, in terms of the house that we have.* | 4/9 |
| *I was getting paid, but I was just burning through all my sick time, so I literally have no sick time left for myself if I ever get sick … my wife was missing 3 days in a week a month, and she wasn’t getting paid for those days.* | 4/7 |

^a^ Patient enrolled in substudy

*MPS II* mucopolysaccharidosis II; *NA* not available
