# Supplementary material for: Caregiver experiences and observations of intrathecal idursulfase-IT treatment in a phase 2/3 trial in pediatric patients with neuronopathic mucopolysaccharidosis II
Source: Orphanet J Rare Dis. 2024 Mar 10;19:110. doi: 10.1186/s13023-024-03034-y (PMC10926613; doi:10.1186/s13023-024-03034-y)
Supplement: Supplementary file 5 — Additional file 5. Table S4. Caregiver descriptions of clinical trial expectations [file 13023_2024_3034_MOESM5_ESM.docx]

**Caregiver experiences and observations of intrathecal idursulfase-IT treatment in a phase 2/3 trial in pediatric patients with neuronopathic mucopolysaccharidosis II**

**Karen S. Yee, Sandy Lewis, Emily Evans, Carla Romano, David Alexanderian**

**Table S4.** Caregiver descriptions of clinical trial expectations

| **Narrative** | **Patient age at trial entry/time of interview, years** |
| --- | --- |
| *We are trying everything and … anything and everything to save him. So, we’re taking any little glimpse of hope we can.* | 2/3^a^ |
| *I just want him to get whatever care he needs and this is going to save his life.* | NA/8 |
| *There is no trajectory for him except down, unless we try anything and everything.* | 3/7 |
| *It’s either do you want the possibility of extending your child’s life, or do you want the certainty of his death? That was it.* | 4/6 |
| *I enrolled him because I could see my son somewhere buried into his body … Like, his personality, I knew it was there and I knew he wanted to come out and show us who he really was, but the disease was sort of holding him back because it was affecting his brain. I knew what the outlook was like for these kids, and I figured if he had any chances of somewhat of a normal life, like I would definitely take it.* | NA/6 |
| *So, we kind of wanted to not watch him regress. We kind of wanted to keep him still learning a little bit.* | 4/7 |
| *I want my child to be able to talk to me. I want him to live. It would allow him to be [himself]. It would allow him not to be shut behind those glass doors of his eyes. It would allow him just to have a voice longer … and that’s worth it.* | 4/7 |
| *Well, the truth is that we were very hopeful, especially in relation with, with the fact the child was going to stop his deterioration … because you saw the evolution of other kids and you thought your child was going to be like them. And since it’s a neurodegenerative disease we thought it was going to stop it, maybe in a more radical way. We thought maybe this is going to save him or so and so.* | NA/8 |
| *I was hoping that he would speak, that we would get his speech back and start to speak because he was so young at the time.* | 3/7 |

^a^ Patient enrolled in substudy

*NA* not available
